# Food access among people who inject drugs in West Virginia

**DOI:** 10.1186/s12954-021-00536-x

**Published:** 2021-08-21

**Authors:** Saba Rouhani, Sean T. Allen, Sara Whaley, Rebecca Hamilton White, Allison O’Rourke, Kristin E. Schneider, Michael E. Kilkenny, Brian W. Weir, Susan G. Sherman

**Affiliations:** 1 Department of Health, Behavior and Society, Bloomberg School of Public Health, Johns Hopkins University, 624 N Broadway, Baltimore, MD 21205 USA; 2 Department of Mental Health, Bloomberg School of Public Health, Johns Hopkins University, Baltimore, MD USA; 3 Department of Health Policy and Management, Bloomberg School of Public Health, Johns Hopkins University, Baltimore, MD USA; 4 DC Center for AIDS Research, Department of Psychology, George Washington University, Washington, DC USA; 5 Cabell-Huntington Health Department, Huntington, WV USA

**Keywords:** Hunger, Food access, Injection drug use, Drug overdose

## Abstract

**Background:**

The substance use epidemic in the United States continues to drive high levels of morbidity and mortality, particularly among people who inject drugs (PWID). Poor access to food often co-occurs with drug use and contributes to associated sequelae, such as risks for HIV and diabetes. The objective of this study was to examine factors associated with adequate food access among PWID in a rural Appalachian community.

**Methods:**

Cross-sectional surveys were used to collect data among PWID aged 18 and older in Cabell County, West Virginia. Frequency of hunger and sociodemographic, structural and drug use characteristics were measured. Adequate food access was defined as reporting ‘never’ going to bed hungry at night in the past six months. Pearson’s *χ*^2^ and *t*-tests and multivariable logistic regression were used to identify factors associated with food access.

**Results:**

Only 71 individuals (17%) reported never going to bed hungry at night in the past six months. Adjusted odds of having adequate food access were higher among PWID who completed high school (aOR 2.94; *P* = 0.010) and usually used drugs alone (aOR 1.97; *P* = 0.025), and lower among PWID who were female (aOR 0.51; *P* = 0.037), experienced homelessness (aOR 0.23, *P* < 0.001), were recently arrested (aOR 0.50 *P* = 0.047), and engaged in receptive sharing of injection equipment (aOR 0.52, *P* = 0.035).

**Conclusions:**

We found extremely low food access in a population of PWID in Appalachia who are vulnerable to overdose and infectious disease transmission. Integrated interventions promoting food access are needed to improve the public health and wellbeing of people who inject drugs in Appalachia.

## Background

The ongoing overdose crisis drives staggering levels of mortality in the United States, with 70,980 deaths recorded in 2019 alone [1]. People who use drugs are also more likely to experience other adverse health outcomes, including HIV, viral hepatitis, cardiovascular disease, and diabetes [2, 3]. The likelihood of experiencing these outcomes is influenced by a constellation of social and economic systems which create a broader risk environment whereby particular populations are more *structurally vulnerable* than others [4, 5]. Structural vulnerabilities, such as lack of sufficient access to food or housing, often co-occur with substance use and amplify risks of experiencing substance use-related morbidity and mortality. Research has shown that people [6, 7] who inject drugs (PWID) are often characterized by multiple structural vulnerabilities and experience a range of adverse health outcomes, including fatal and non-fatal overdose and injection-associated HIV and viral hepatitis acquisition [8, 9].

Lack of food security, characterized by ‘limited or uncertain availability of nutritionally adequate and safe foods, or limited or uncertain ability to acquire acceptable foods in socially acceptable ways [10] is linked to a myriad of physical, socioeconomic and mental health vulnerabilities. A growing body of literature has described the syndemic overlap between hunger, injection drug use, and HIV risk [11]. Biologically, nutritional insufficiency impairs immune responses, increasing susceptibility to and severity of infectious diseases, including HIV [11, 12]. Experiencing even moderate food insecurity is associated with poorer mental health outcomes [13, 14] and barriers to healthcare, which result in lower uptake of prevention and treatment services for both HIV and substance use [15]. Further, the need to obtain food can constitute a competing need among marginalized individuals, leading to deprioritization of other protective behaviors [16]. PWID struggling to access food are indeed more likely to engage in behaviors (e.g., syringe sharing, condomless sex) that increase risks for HIV acquisition [15, 17–20].

In the United States, the prevalence of food insecurity exceeds those found in other comparable high-income countries [21], and this burden is disproportionately concentrated among marginalized populations such as PWID [15]. Studies in North America suggest that between 30–50% of PWID have some level of food insecurity [15], and that they have poorer nutritional indicators than both non-injecting drug using and non-drug-using populations [22]. However, most of these estimates have been generated in urban centers, with little data available from rural drug-using populations. Rural Appalachian states face a dual burden of food insecurity and substance use. West Virginia is at the center of this crisis, with 15% prevalence of food insecurity [23] among the state’s general population and the highest age-adjusted rate of overdose fatalities in the country [24]. High rates of substance use, transitions to injection drug use, and limited access to sterile injection equipment across the state have been highlighted as drivers of emerging HIV clusters [25]. Further, recently instated requirements for food assistance programs in West Virginia limit eligibility to individuals with employment, which is likely to disproportionately restrict access among PWID given lower levels of formal employment [26, 27]. Despite these overlapping burdens, there is a paucity of literature exploring food security among rural PWID in Appalachia.

To address this gap, we sought to estimate the prevalence of PWID with adequate access to food in Cabell County, West Virginia, a county that is 86% rural and has an estimated 2.4% prevalence of recent injection drug use [28]. While the broader construct of food security encompasses qualitative measures of nutritional adequacy, as well as psychosocial factors determining stable food supply [12], we focus on a quantitative aspect of food security defined by the frequency of persons going to bed at night hungry because there was not enough food, referred to here as food access. We examined factors associated with adequate food access among this population in order to inform potential actionable strategies to ensure the basic needs of rural PWID are met.

## Methods

### Study design and recruitment

Data were collected as part of the West Virginia COUNTS! Study which was conducted in June-July 2018 to estimate the number of PWID in Cabell County, West Virginia. Details of this study are available elsewhere [28]; briefly, capture-recapture methodology was employed for population estimation, comprising two rounds of recruitment. Participants were enrolled in the study at the harm reduction program at the Cabell-Huntington Health Department and throughout Cabell County in public parks, parking lots, and other public spaces where PWID were known to congregate. Anonymous surveys were administered using audio computer assisted self-interviewing (ACASI) software on tablets with headphones. Due to high levels of injection drug use-associated stigma, broad inclusion criteria were utilized: having ever used drugs and at least 18 years of age. Participants were given snack bags or $10 grocery gift cards as incentives. This study was approved Johns Hopkins Bloomberg School of Public Health Institutional Review Board. The analytic sample for the current study was comprised of participants reporting injection drug use in the past 6 months (*n* = 420).

### Outcome

The primary outcome of interest for this analysis was adequate food access. To approximate this outcome, participants were asked, “In the past 6 months, how often did you go to sleep at night hungry because there was not enough food?” We employed this single-item measure for comparability with similar populations in urban areas [29]. Ordinal categorical response options included: Every day; 2–6 days per week; at least once a week; once a month; less than once a month; never; refuse to answer; don’t know. After consideration of the distribution of our responses and existing literature demonstrating the psychological and physical implications of experiencing even moderate levels of hunger [13, 15, 30], we dichotomized the outcome variable and defined adequate food access as ‘never’ going to bed hungry in the past six months.

### Sociodemographic variables and structural vulnerabilities

Age was measured and analyzed as a continuous variable. Gender (male vs female) and relationship status were dichotomized (in a relationship or married vs single). Participants were asked to “select all that apply” for their racial and ethnic identities with options including Black, White, Asian, Pacific Islander, American Indian/Alaskan Native, and multiracial, and if they identified as Hispanic. Due to the low prevalence of racial/ethnic identities other than non-Hispanic, White, we collapsed these measures into non-Hispanic White vs all other racial/ethnic identities. PWID identifying as gay, lesbian, bisexual, or any other sexual orientation other than ‘heterosexual or straight’ were classified as sexual minorities. Educational attainment was defined as having a high school diploma, GED or higher (e.g., graduate studies) versus not graduating high school. Housing status was defined as considering oneself homeless. Unemployment, recent (past 6 month) arrest, and currently having health insurance were measured and analyzed as dichotomous variables. Oral, vaginal or anal sex exchanged for money, food, drugs or favors in the past 6 months was classified as engaging in transactional sex work.

### Substance use variables

Substance use variables were ascertained by asking about behaviors occurring in the past six months. Number of daily injections (“How many times do you inject drugs on a typical day?”) was measured continuously with values ≥ 50 recoded as missing (*n* = 2). Participants were asked their earliest age of injection, and ages were dichotomized to classify individuals who began injecting as minors. Receptive sharing of injection equipment was defined as reusing syringes or other injection equipment (cookers, cotton, or rinse water) known to have been used by somebody else. Participants reported where they usually used drugs, with public drug use defined as in a stairwell of a building or business, abandoned building, vehicle, public bathroom, park or another green space, or on the street [31]. Solitary drug use was defined as typically used drugs alone. Overdose was measured as “having overdosed to the point of passing out [in the past 6 months].” Consumption and route of administration of specific drugs (heroin, opioid pain relievers, crack, cocaine, fentanyl, and crystal methamphetamine) were measured by asking: “Have you [smoked/injected/inhaled or snorted/swallowed] [specific drug] in the past 6 months?” After comparison and observed parity between injection-specific drug consumption and consumption by any route, results were aggregated to reflect use of each substance by any route.

### Statistical analysis

Descriptive comparisons of variables by food access were conducted using Pearson’s *χ*^2^ and Fisher’s exact tests (where cell sizes were < 10) and t-tests for continuous variables. Univariable and multivariable logistic regression was used to estimate crude and adjusted odds of adequate food access, respectively. Variables were considered for inclusion based on prior relevant literature [19, 29] and evidence of an association (*P* < 0.2) at the bivariate level. Unemployment and public drug use were excluded due to high collinearity with experiencing homelessness. Sex work was highly correlated with gender and, given insufficient sample size to assess statistical evidence of interaction, it was excluded from the final model. After inclusion in the initial adjusted model, several nonsignificant variables (relationship status, daily injections, overdose history and drugs used) were removed to achieve parsimony and improve model fit. Models were compared using post-estimation Wald tests and fit statistics (AIC/BIC/log likelihood). Homelessness (instead of public drug use or unemployment) and gender (instead of sex work) were retained as these variables yielded the best fit statistics. A Hosmer–Lemeshow goodness-of-fit test suggested adequate fit of the final model (*P* = 0.84). All analyses were conducted in Stata v15 (StataCorp, College Station, TX).

## Results

### Description of the analytic sample

Most of our participants were male (61%) and identified as non-Hispanic White (84%; Table 1). Participants ranged from 19 to 63 years old, with an average of 35.8 years old. Nearly half were in a relationship (46%) and 17% identified as a sexual minority. The majority of participants reported having completed at least a high school education (73%) and currently having health insurance (73%). However, markers of socioeconomic and structural vulnerability were prevalent. The majority were homeless (56%) and unemployed (66%). Eighteen percent reported engaging in transactional sex in the past 6 months, the majority of whom (64%) were women (data not shown). One third (34%) had been arrested in the past 6 months.

**Table 1:** Characteristics of people who have injected drugs in the past 6 months (*n* = 420) by food access

	Total (*N* = 420)	Inadequate food access (*n* = 349)	Adequate food access (*n* = 71)	*χ*^2^*P* value
	*n* (col %)	*n* (col %)	*n* (col %)	
*Sociodemographic data*				
Age, mean (SD)	35.8 (8.5)	35.9 (8.6)	35.4 (8.2)	0.665
Gender				
Male	257 (61.2)	207 (59.3)	50 (70.4)	
Female	163 (38.8)	142 (40.7)	21 (29.6)	0.080
Race/ethnicity				
White, non- Hispanic	341 (83.6)	283 (83.5)	58 (84.1)	
Other	67 (16.4)	56 (16.5)	11 (15.9)	0.906
Sexual minority	73 (17.4)	63 (18.1)	10 (14.1)	0.416
In a relationship	193 (46.2)	155 (44.7)	38 (53.5)	0.173
*Structural factors*				
Educational attainment	304 (72.6)	241 (69.3)	63 (88.7)	***0.001***
Health insurance	305 (72.6)	251 (71.9)	54 (76.1)	0.476
Homelessness	235 (56.0)	218 (62.5)	17 (23.9)	***0.000***
Unemployed	277 (66.0)	237 (67.9)	40 (56.3)	0.061
Arrest	141 (33.6)	125 (35.8)	16 (22.5)	***0.031***
Engaged in transactional sex	77 (18.3)	69 (19.8)	8 (11.3)	0.096
*Substance use*				
Mean number of daily injections	4.4 (3.9)	4.5 (4.0)	3.6 (3.5)	0.064
Receptive sharing of injection equipment	257 (61.2)	230 (65.9)	27 (38.0)	***0.000***
Began injecting as a minor	83 (19.8)	68 (19.5)	15 (21.1)	0.751
Public drug use	199 (47.4)	178 (51.0)	21 (29.6)	***0.001***
Use drugs alone	133 (31.7)	94 (26.9)	39 (54.9)	***0.000***
Experienced overdose, past 6 m	179 (42.6)	158 (45.3)	21 (29.6)	***0.015***
Drugs used (any route)				
Heroin	352 (83.8)	296 (84.8)	56 (78.9)	0.215
Pain relievers (pills)	179 (42.6)	155 (44.4)	24 (33.8)	0.099
Fentanyl	233 (55.5)	202 (57.9)	31 (43.7)	***0.028***
Cocaine	204 (48.6)	171 (49.0)	33 (46.5)	0.699
Speedball (injection)	161 (38.3)	140 (40.1)	21 (29.6)	0.096
Crack	228 (54.3)	194 (55.6)	34 (47.9)	0.235
Crystal methamphetamine	315 (75.0)	268 (76.8)	47 (66.2)	0.060

Significant* p*-values (*P* < 0.05) are shown in bold italics

All measures refer to exposure in the past 6 months, except for sociodemographic factors and education level

### Substance use

PWID reported injecting an average of 4 times daily, and the majority (61%) engaged in receptive sharing of injection equipment in the past 6 months. A fifth (20%) began injecting before the age of 18. Just under half (47%) of participants reported that they most commonly used drugs in public spaces, while 32% usually used drugs alone. A quarter of the sample had experienced at least one overdose in the past 6 months.

Drugs commonly used in the past 6 months, by any route of administration, were heroin (84%), crystal methamphetamine (75%), fentanyl (56%), cocaine (56%), crack (54%), and opioid pain relievers (43%). A similar distribution was observed among injected drugs, with participants most commonly reporting injection of heroin (81%), crystal methamphetamine (71%), fentanyl (55%), speedball (cocaine and heroin together; 38%), cocaine (34%), and opioid pain relievers (23%).

### Food access among PWID

**Fig. 1:**
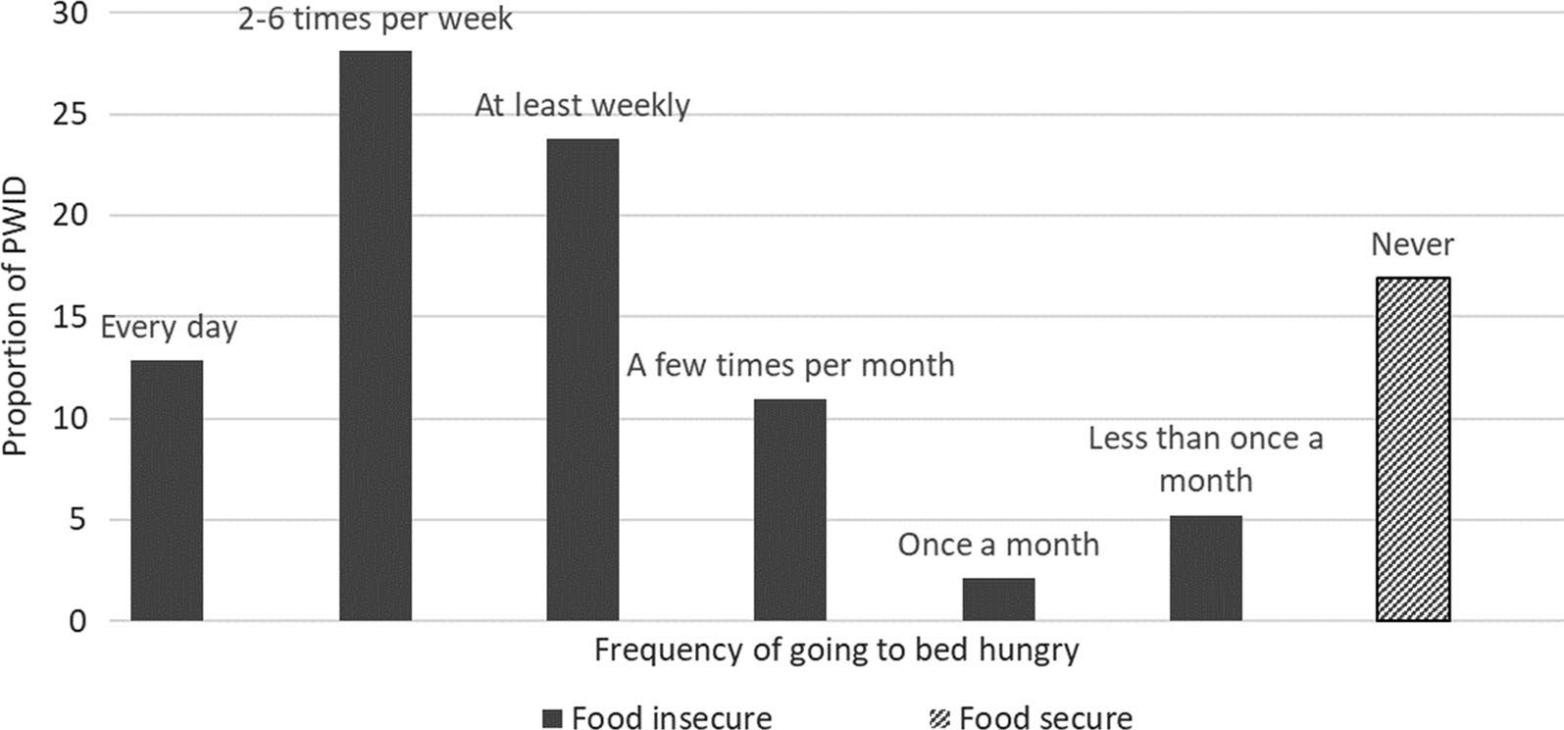
Proportion of people who inject drugs who reported going to bed hungry in the past 6 months in West Virginia (*n* = 420)

Only 71 individuals (17%) had not gone to sleep hungry in the past 6 months, which is how we operationalized adequate food access. Participants who reported going to sleep hungry most commonly indicated that this happened at least weekly (24%), 2–6 times a week (28%), or everyday (13%) (Fig. 1).

A greater proportion of PWID with adequate food access had higher educational attainment (89% vs 69%; *p* = 0.001) and reported using drugs alone (55% vs 27%; *P* < 0.001); in contrast, a greater proportion of PWID without adequate food access were experiencing homelessness (24% vs 63%; *P* < 0.001), recently arrested (23% vs 36%; *P* = 0.031) or experienced overdose (30% vs 45%; *P* = 0.015), reported receptive sharing of injection equipment (38% vs 66%; *P* < 0.001), and engaged in public drug use (30% vs 51%; *P* = 0.001). The only substance significantly associated with adequate food access at the bivariate level was fentanyl; 44% of persons with adequate food access reported recent fentanyl use, compared to 58% of those who did not.

### Correlates of adequate food access

Crude and adjusted odds ratios for variables included in the final model are shown in Table 2. Factors independently associated with greater odds of adequate food access in the multivariable logistic regression model included educational attainment (aOR 2.94; *P* = 0.010) and usually using drugs alone (aOR 1.97; *P* = 0.025). Factors independently associated with reduced odds of adequate food access included being female (aOR 0.51; *P* = 0.037), experiencing homelessness (aOR 0.23, *P* < 0.001), recent arrest (aOR 0.50 *P* = 0.049), and receptive sharing of injection equipment (aOR 0.52, *P* = 0.035). After adjustment for sociodemographic and structural factors, no specific drug was associated with increased or decreased odds of food access. A goodness of fit test indicated adequate model fit (*P* value = 0.838).

**Table 2:** Results of a multivariable logistic regression model estimating odds of adequate food access among people who inject drugs (*n* = 420)

	Unadjusted odds of adequate food access			Adjusted odds of adequate food access		
	uOR	95% CI	*P* value	aOR	95% CI	*P* value
*Sociodemographic data*						
Age (years)	0.99	0.96–1.02	0.664	0.97	0.94–1.00	0.053
Gender						
Male	Reference			Reference		
Female	0.61	0.35–1.06	0.082	0.51	0.27–0.96	***0.037***
*Structural factors*						
Educational attainment	3.50	1.62–7.55	***0.001***	2.94	1.29–6.69	***0.010***
Considers self homeless	0.19	0.11–0.34	***0.000***	0.23	0.12–0.44	***0.000***
Arrest	0.52	0.29–0.95	***0.033***	0.50	0.26–0.99	***0.047***
*Substance use*						
Receptive sharing of injection equipment	0.32	0.19–0.54	***0.000***	0.52	0.29–0.96	***0.035***
Usually uses drugs alone	3.31	1.96–5.58	***0.000***	1.97	1.09–3.57	***0.025***

Significant* p*-values (*P* < 0.05) are shown in bold italics

All measures refer to exposure in the past 6 months, except for sociodemographic factors and education level

## Discussion

This study reports extremely low prevalence of adequate food access among PWID in a rural Appalachian community grappling with disproportionately high rates of substance use and overdose. In Cabell County, where an estimated 2.4% of the population injects drugs [28], we found that only 17% of PWID were likely to report adequate food access. This prevalence is much lower than estimates of food security in the general population (88%) [32] and considerably lower than populations of PWID from North American studies in California (38%- 42%) [19] Ontario (45%) [15], and Vancouver (35%) [17, 33, 34]. However, meaningful comparisons are hampered by several factors, including that the majority of injection drug use-related literature reflects urban populations as well as varying definitions of food access and security across studies. Nonetheless, these data highlight the extent of unmet need for food, a basic requirement for health and survival, among vulnerable individuals with multiple competing health needs.

Consistent with existing literature [22, 33–36], there was an inverse relationship between food access and sharing injection equipment. While the direction of this association cannot be inferred from cross-sectional analyses, this finding highlights the co-occurrence of hunger and HIV risk behaviors which may act to reinforce one another and compound risks of HIV acquisition. Food insecurity is linked to both increased susceptibility to HIV infection, and poorer adherence to pre-exposure prophylaxis [37, 38] and anti-retroviral therapy [33, 39], underscoring the importance of food access for initiatives aimed at preventing HIV acquisition among PWID. More broadly, basic subsistence needs such as access to food and housing serve as competing priorities [40, 41] that drive lower uptake of prevention and treatment among people who are vulnerable to or living with HIV [42, 43], thereby compounding their risks of negative health outcomes [16]. These factors are particularly relevant in the context of Cabell County, which is among a growing list of counties experiencing increases in injection-associated HIV incidence in recent years [44–47]. Interventions to promote access to sufficient quantity and quality of food for PWID may be complementary to HIV prevention and substance use services in these settings. Various global health initiatives integrating food security and nutrition interventions with HIV/AIDS programs in lower and middle-income countries exist; however, a recent review [48] highlighted that a paucity of evidence and best practices to achieve this in the United States. Despite growing calls to pursue food provision as an important harm reduction strategy among PWID, the drug treatment or syringe services programs in North America that do offer food services are highly variable and there remain no guidelines to inform best practices or implementation at scale [49]. These data further underscore the need for interventions promoting food access to be available for people at risk of and living with HIV in rural settings, including PWID.

Another important finding from this study was that women who inject drugs had significantly lower odds of having sufficient food access than their male counterparts. This may be due to several factors related to gender roles and disparities. Women in our study more frequently engaged in transactional sex. Research demonstrates low food security among women who sell sex, and this has been shown to reinforce their need to engage in sex work and reduce their negotiating power in terms of utilizing HIV prevention measures with clients, such as condom use [20, 22, 29]. However, this population often has a high prevalence of overlapping structural vulnerabilities, including homelessness, making specific effects difficult to decipher. Interactions between sex work, gender, and food access should be further explored in studies with sufficient sample size to inform targeting of interventions promoting food security. Women are also more likely than men to be responsible for children, increasing their financial burdens. Lower food access may therefore reflect the stretching of limited resources among women with their dependents [20, 46, 50], and introduce even greater incentive for women to prioritize food acquisition over other health needs [16, 37, 38]. In West Virginia, 42–52% of households with children utilize SNAP benefits [51] and national data demonstrate that single mother households have the lowest rate of food security in the country [29]. There is a well-established body of evidence demonstrating the importance of food security and nutritional sufficiency for women’s reproductive health and the subsequent health of their children [52]. Bolstering food access may therefore represent a useful and high-impact entry point for averting downstream health risks among a vulnerable sub-population of women who use drugs.

Recent arrest was associated with lower odds of adequate food access among our sample, but the direction of this association cannot be inferred with the available data. Individuals with low access may be arrested for crimes related to their hunger and fundamental survival (e.g., food theft); alternately, arrests may lead to financial costs (e.g. bail, legal fees) and interrupt stabilizing forces, which in turn can impact food access. For example, loss of employment due to arrest may indirectly lead to reduced access to food, particularly if there are work requirements for food assistance as in West Virginia. Evidence also suggest that arresting people with substance use disorders can interrupt access to treatment and result in higher-risk substance use and overdose [53, 54]. Taken together, this suggests that PWID with recent arrests may be particularly vulnerable to both food insecurity and drug-related harms; efforts should therefore be made to ensure that all PWID, regardless of interaction with the justice system, have consistent and low-threshold access to food.

We identified several factors associated with food access among PWID in rural Appalachia that illustrate a portrait of pervasive structural vulnerability. Rather than being specific to substances used, food access was related to factors such as education and housing. This is consistent with the broader literature, which highlights structural markers of poverty (e.g., lack of housing [17, 19, 20, 29, 33, 34, 39], education [29] and employment [55, 56]) as the most strongly and consistently associated with hunger. In the state of West Virginia, prevalence of these indicators in the general population is higher than the United States’ average; in 2019, for example, 18% of the WV population were living in poverty [57] compared with 12% nationally, and the unemployment rate was 5% relative to 3.5% nationally [58]. Data specifically among people who inject drugs throughout the state are not available, to our knowledge. In this study sample, however, we observed an unemployment rate of 66%, in stark contrast to state-wide levels among the general population. Further, since measures to reduce the spread of the novel SARS-COV-2 (COVID-19) virus began in March 2020, the overall unemployment rate in West Virginia immediately increased to 15% [59], and there have been dramatic increases in unmet need for food and SNAP applications nationally [60, 61]. Other supportive services, such as school feeding programs, are struggling to meet demand [62] and it is estimated that over 20% of children in the state are food insecure [63]. As such, the proportion of PWID, and specifically women with children, who are at risk of hunger in this setting is likely to be even higher than estimates provided here.

Taken together, these data suggest that integrated programs designed to address multiple, overlapping vulnerabilities are needed to respond to the crisis of food insecurity in this population. Contextualizing results in West Virginia’s policy context highlights areas where feasible policy changes could be implemented to mitigate food insecurity and HIV risks alike. For example, strict work requirements for food assistance, relative to other states, represent a clear obstacle to promoting wider food access in marginalized populations and may help to explain the comparatively low estimates provided here.

Results should be viewed in light of several limitations. Measures of food access and security vary across studies and have not been standardized in this population, and self-report data can be dependent on the individual’s perception [19]. Further, our outcome was captured using a single item measure to compare with recent associated literature in an urban center, and for ease of survey administration among this hard-to-reach population. While this approximated the quantitative component of food security, differences in measurement tools should be considered when comparing our estimates to those in other studies. Given the trauma associated with hunger, and the ways trauma is also a driver of substance use itself and related risk behaviors [64], we adopted a strict cutoff classifying any recent experiences of going to bed hungry as counter to having adequate food access. This is further supported by prior literature suggesting that moderate definitions of experiencing hunger may be more sensitive than extreme or severe ones [65, 66]. We also posited that any recent experience of uncertainty about securing one’s next meal could comprise a tangible competing priority potentially influencing HIV risk. Nonetheless, comparison with other studies should be made keeping the lack of consistency between metrics of food security and access, particularly in this population, in mind. Results should therefore be viewed with full consideration of the differences between our metric and other published estimates. We lacked an adequate sample size to explore potentially meaningful interactions, e.g., between sex work, gender, and food access. We also did not have data on pregnancy or motherhood, limiting our ability to more fully explore whether the relationship between adequate food access and gender was related to these factors. Data on nutritional indicators (e.g. underweight, specific micronutrient deficiencies, overweight/obesity) and food assistance (e.g., proportion enrolled in SNAP) were also not collected. While we explored a range of drug use via any route of administration, results should not be extrapolated to the broader population of PWUD given the parent study’s focus on injection drug use and the restriction to PWID within this analysis. Finally, as a cross-sectional study, we are unable to make inferences regarding causality or the direction of associations.

## Conclusions

We report extremely low food access and numerous intersecting structural vulnerabilities among PWID, particularly women, in rural West Virginia. Data presented illustrate that few basic needs among this population were being met even prior to the COVID-19 pandemic and that this coincided with riskier practices that increase the likelihood of public health outbreaks such as HIV and overdose. Amid the current crisis, designing and evaluating comprehensive interventions to promote food provision as harm reduction in this population will be important for mitigating negative social and health outcomes in rural Appalachia.

## Data Availability

Data are not publicly available to protect participant anonymity.

## Abbreviations

ACASI: Audio computer-assisted self-interview
HIV: Human immune-deficiency virus
PWID: People who inject drugs
SNAP: Supplemental Nutrition Assistance Program
